# Serum Fatty Acid-Binding Protein 4: A Potential Diagnostic Marker Linking Lipid Metabolism and Inflammation in Intrahepatic Cholestasis of Pregnancy

**DOI:** 10.3390/diagnostics16040525

**Published:** 2026-02-10

**Authors:** Sadun Sucu, Sadullah Özkan, Mustafa Alperen Aksan, Murat Levent Dereli, Belgin Savran Üçok, Ramazan Erda Pay, Kemal Sarsmaz, Harun Egemen Tolunay, Ali Turhan Çağlar

**Affiliations:** 1 Ankara Etlik City Hospital, Ankara 06170, Turkey; 2 Ankara Etlik Zübeyde Hanım Hospital, Ankara 06170, Turkey; 3 Manisa Celal Bayar University Hafsa Sultan Hospital, Manisa 45030, Turkey; 4 Private Clinic, Ankara 06680, Turkey

**Keywords:** adipokine, biomarker, FABP4, intrahepatic cholestasis of pregnancy, lipid metabolism

## Abstract

**Objective:** This study aimed to investigate maternal serum fatty acid-binding protein 4 (FABP4) levels in pregnancies complicated by intrahepatic cholestasis of pregnancy (ICP) and to evaluate its diagnostic and prognostic utility for maternal and neonatal outcomes. **Methods:** This prospective case–control study included 44 women diagnosed with ICP and 44 gestational age-matched healthy pregnant controls between 24 and 41 weeks of gestation. Serum FABP4 concentrations were measured using a quantitative sandwich enzyme-linked immunosorbent assay (ELISA). Demographic, biochemical, and perinatal data were collected prospectively. Group comparisons were performed using the *t*-test or Mann–Whitney U test, correlations by Pearson or Spearman tests, and diagnostic performance by receiver operating characteristic (ROC) curve analysis. **Results:** Maternal serum FABP4 levels between 25 and 39 weeks of gestation were significantly higher in the ICP group than in the control group (median 3.60 [Q1–Q3: 3.25–4.20] vs. 2.40 [Q1–Q3: 2.00–2.95] ng/mL; *p* < 0.001). ROC analysis revealed excellent diagnostic accuracy for ICP (AUC = 0.899; 95% CI: 0.816–0.953; *p* < 0.001) with an optimal cut-off value of >3.0 ng/mL, yielding 90% sensitivity and 84% specificity. FABP4 correlated inversely with gestational age at delivery in the total cohort (*r* = −0.430, *p* < 0.001) but not within the ICP subgroup. In predicting composite neonatal outcomes, FABP4 showed moderate performance (AUC = 0.634, 95% CI: 0.525–0.734, *p* = 0.032) and limited predictive ability within the ICP group (AUC = 0.535, *p* = 0.685). **Conclusions:** Maternal FABP4 levels are significantly elevated in ICP and show high diagnostic accuracy for ICP but have limited prognostic value for neonatal outcomes. FABP4 may represent a novel biomarker reflecting the metabolic–inflammatory interplay underlying the pathophysiology of intrahepatic cholestasis of pregnancy.

## 1. Introduction

Intrahepatic cholestasis of pregnancy (ICP) is the most common liver disorder unique to pregnancy, characterized by the onset of pruritus in late gestation, along with abnormal liver enzyme levels and elevated circulating bile acids [1]. Although ICP is usually reversible after delivery, it is associated with significant maternal and fetal risks, including preterm delivery, meconium-stained amniotic fluid, intrauterine growth restriction, and stillbirth [2,3]. Despite its clinical relevance, the etiology of ICP remains incompletely understood. It is likely multifactorial, and includes genetic susceptibility, environmental influences, and hormonal changes. Estrogen and progesterone metabolites are involved in the pathogenesis by modulating bile acid transport and signaling through nuclear receptors, leading to impaired excretion of bile acids [4].

In recent years, attention has increasingly focused on the role of adipokines and lipid-binding proteins in pregnancy-related disorders. Fatty acid-binding proteins (FABPs) are a family of small intracellular lipid chaperones that facilitate fatty acid transport and are involved in metabolic and inflammatory processes [5]. Fatty acid-binding protein 4 (FABP4, also known as adipocyte FABP or aP2) is highly expressed in adipocytes, macrophages, endothelial cells, and trophoblasts [6]. FABP4 regulates lipid metabolism, insulin sensitivity, and inflammatory signaling, and aberrant circulating levels are associated with obesity, metabolic syndrome, type 2 diabetes, and atherosclerosis [7,8].

Changes in maternal FABP4 concentrations have been documented in the context of pregnancies complicated by metabolic and hypertensive disorders, including gestational diabetes mellitus and preeclampsia. Elevated FABP4 levels have been observed in women with preeclampsia both at diagnosis and before the onset of the disease, indicating a possible predictive role [6,9]. Similarly, FABP4 levels are elevated in maternal and cord blood in GDM, and correlate with insulin resistance and adverse fetal outcomes [10]. These findings support the hypothesis that FABP4 may act as a biomarker reflecting the metabolic and inflammatory milieu of pregnancy complications. In addition, FABPs have been experimentally shown to interact with peroxisome proliferator-activated receptors (PPARs), nuclear receptors that control lipid metabolism, inflammation, and placental function. This interaction may represent a mechanistic link between dysregulated fatty acid signaling and pregnancy pathologies [11].

Considering that ICP shares several features with metabolic disorders, including altered bile acid homeostasis and impaired lipid metabolism, which may contribute to compromised outcomes in the perinatal period, the potential role of FABP4 in its pathophysiology should be investigated. To date, no study has systematically examined maternal serum FABP4 levels in ICP. The identification of novel biomarkers such as FABP4 could improve early diagnosis, risk stratification, and understanding of disease mechanisms, and ultimately lead to targeted interventions to improve maternal and fetal outcomes.

## 2. Materials and Methods

### 2.1. Study Design and Participants

**Figure 1:**
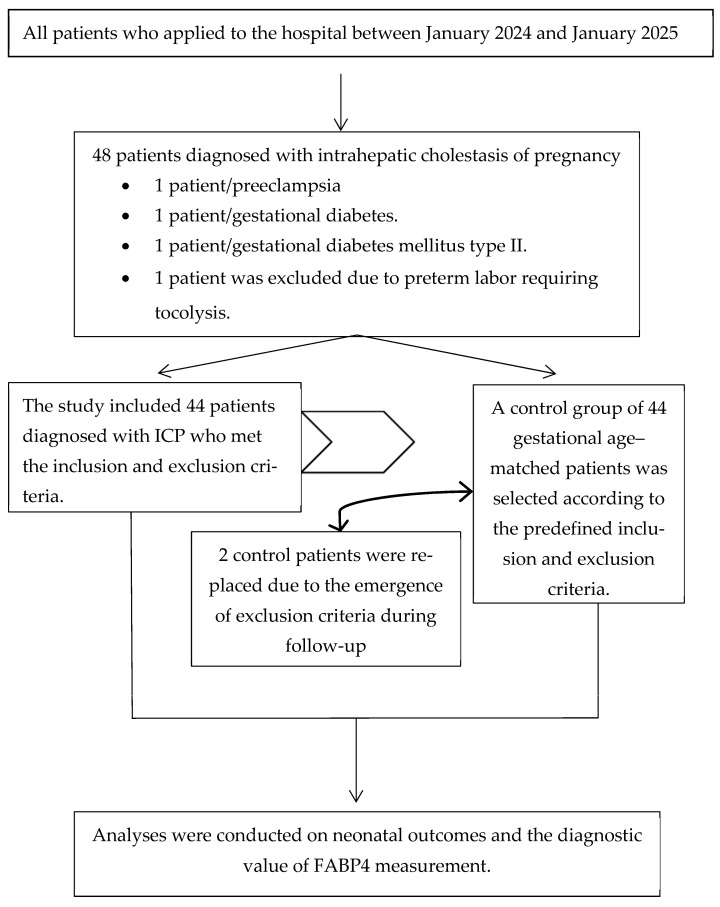
Flowchart.

The study included pregnant women aged 18 to 45 years with a singleton pregnancy who were admitted to the perinatology clinic of Etlik City Hospital between 24 and 41 weeks of gestation. The case group (*n* = 44) consisted of women diagnosed with ICP based on clinical symptoms such as pruritus, along with elevated serum bile acid levels and/or abnormal liver function tests. The control group (*n* = 44) consisted of healthy pregnant women without hepatic or systemic disease. Women with multiple pregnancies, maternal systemic disease (including chronic liver, renal, endocrine, or cardiovascular disease), fetal congenital or chromosomal abnormalities, or those taking chronic medications were excluded. Additionally, participants who had received antenatal corticosteroids or tocolytics prior to blood sampling were excluded (Figure 1). Written informed consent was obtained from all participants before enrollment in the study.

### 2.2. Sample Collection and Storage

Venous blood samples (2 mL) were collected from each participant during routine diagnostic testing. Blood samples for FABP4 were collected only once. Serum was separated by centrifugation at 1000× *g* for 20 min at 4 °C and stored in aliquots at −80 °C until analysis. To minimize pre-analytical variability, hemolyzed samples and those subjected to repeated freeze–thaw cycles were excluded.

### 2.3. Measurement of FABP4

Serum FABP4 levels were measured using a commercially available quantitative sandwich enzyme-linked immunosorbent assay (ELISA) kit (SEB693Hu; Cloud-Clone Corp., Wuhan, China) in strict accordance with the manufacturer’s protocol. Briefly, 100 μL of standards, blanks, and appropriately diluted serum samples were dispensed in duplicate into pre-coated 96-well microplates and incubated at 37 °C for 60 min. After incubation, unbound components were removed, and 100 μL of Detection Reagent A was added to each well and incubated for 1 h, followed by three washes. Then, 100 μL of Detection Reagent B was added and incubated at 37 °C for 30 min. After five washes, 90 μL of tetramethylbenzidine (TMB) substrate solution was added, and the plates were incubated in the dark for 10–20 min. The reaction was stopped by adding 50 μL of stop solution, and optical density was immediately measured at 450 nm using a microplate reader (BioTek Instruments, Winooski, VT, USA). The assay had a detection range of 0.156–10 ng/mL, with a minimum detectable concentration of <0.059 ng/mL. Intra-assay and inter-assay coefficients of variation (CVs) were <10% and <12%, respectively, ensuring the reliability of the measurement.

### 2.4. Clinical and Laboratory Parameters

Demographic variables (age, parity, gravidity, BMI, obstetric history), routine biochemical parameters (AST, ALT, bile acids), and perinatal outcomes (delivery mode, APGAR scores, admission to neonatal intensive care unit, birth weight, fetal distress) were prospectively collected using standardized data collection forms.

### 2.5. Statistical Analysis

Statistical evaluation was carried out using Jamovi statistical software (version 2.6.44), an open-source analysis program. Data normality was assessed using graphical techniques, including histograms and normal probability plots, as well as formal tests such as the Kolmogorov–Smirnov and Shapiro–Wilk tests. Continuous variables with a normal distribution were reported as mean ± standard deviation and analyzed with the independent samples *t*-test. Variables not normally distributed were expressed as median with quartiles (Q1–Q3), and intergroup comparisons were made using the Mann–Whitney U test. Categorical data were presented as counts and percentages. Associations between categorical variables were evaluated using the chi-square test, and Fisher’s exact test was used when expected frequencies were too low for the chi-square test assumptions. Associations between continuous variables were evaluated using Pearson correlation analysis when the data followed a normal distribution, and Spearman rank correlation analysis was used for variables with non-normal distributions. The predictive ability of maternal FABP4 levels for composite neonatal outcomes and intrahepatic cholestasis of pregnancy (ICP) was assessed using receiver operating characteristic (ROC) curve analysis. After determining the optimal cut-off value, diagnostic performance metrics, including sensitivity, specificity, and area under the curve (AUC), were calculated. A *p* value below 0.05 was considered statistically significant.

### 2.6. Power Analysis

For the power analysis in this study, the study by Wang et al., which evaluated FABP4 levels in the groups, was used to calculate the effect size [12]. With an effect size of 10.33, an alpha error of 0.05, and a power (1-beta) of 95%, the minimum required number of patients per group was 17. However, considering the evaluation of neonatal outcomes, the possibility of non-parametric data distribution, and the need for correlation analyses, the sample size was increased by 150%, resulting in 44 patients in each group. The post hoc power analysis showed a power value above 95%.

## 3. Results

The study population consisted of 88 pregnant women, divided equally into two groups: 44 women diagnosed with intrahepatic cholestasis of pregnancy (ICP) and 44 women with uncomplicated pregnancies serving as controls. Maternal blood samples were collected between 25 and 39 weeks of gestation. The median gestational age at sample collection was 35 weeks in both groups. As shown in Table 1, there were no statistically significant differences between the ICP and control groups in maternal age, body mass index at sampling, gestational weight gain, gravidity, history of miscarriage, or gestational age at blood collection (all *p*-values > 0.05). However, parity was significantly lower in the ICP group (*p* = 0.031). Both systolic and diastolic blood pressure were significantly higher in the ICP group compared to the control group (*p* < 0.001 and *p* = 0.008, respectively).

Hematological parameters, including white blood cell count, hemoglobin, hematocrit, platelet count, neutrophil count, and lymphocyte count, were similar between groups (*p* > 0.05 for all). As expected, liver function tests showed significantly increased ALT and AST levels in ICP patients compared to controls (*p* < 0.001 for both). In addition, fibrinogen was significantly higher (571 vs. 453 mg/dL, *p* < 0.001), while INR was modestly but significantly elevated in ICP patients (0.92 vs. 0.89, *p* = 0.007). The Endothelial Activation and Stress Index (ESAIX), calculated by dividing the product of blood serum LDH and creatinine levels by the platelet count, indicates increased endothelial activation with higher values [13]. Although the ICP group had higher values, there was no statistically significant difference between the groups. Other biochemical markers, including creatinine, BUN, fasting glucose, PT, and aPTT, did not differ significantly between groups. Maternal serum concentrations of FABP4 were significantly higher in the ICP group compared to healthy pregnancies (median 3.60 ng/mL [Q1–Q3 3.25–4.20] vs. 2.40 ng/mL [Q1–Q3 2.00–2.95], *p* < 0.001; Table 2). When stratified by gestational age at blood sampling, maternal serum FABP4 levels remained consistently higher in ICP patients in all subgroups (<34 weeks, 34–37 weeks, and ≥37 weeks) compared to controls (Table 2).

As shown in Table 3, delivery occurred significantly earlier in ICP pregnancies compared to controls (median 37 vs. 39 weeks, *p* < 0.001). Birth weight was also lower in the ICP group (2775 g vs. 3100 g, *p* = 0.002). One-minute APGAR scores were significantly lower in ICP pregnancies (*p* = 0.036), while five-minute scores did not differ between groups.

There were no differences in the mode of delivery (cesarean vs. vaginal) or in the distribution of infant sex. Neonates born to ICP mothers were admitted to the neonatal intensive care unit (NICU) more frequently (15.9% vs. 4.5%), but this difference was not statistically significant (*p* = 0.157). Poor composite neonatal outcomes (CNO)—defined as preterm birth before 34 weeks, low birth weight (<2500 g), NICU admission, or a 5 min APGAR score < 7—were significantly more common in the ICP group compared to controls (27.3% vs. 6.8%, *p* = 0.023; Table 3).

As shown in Table 4, FABP4 levels did not correlate significantly with maternal age, BMI, weight gain, parity, neonatal birth weight, or APGAR scores (*p* > 0.05 for all). However, in the entire study population, FABP4 levels showed a significant negative correlation with gestational age at delivery (*r* = −0.430, *p* < 0.001). This relationship was not observed when analyses were restricted to the ICP subgroup (*r* = −0.109, *p* = 0.483). No significant correlation was found between FABP4 levels and gestational age at the time of blood sampling in either group (ICP: *r* = 0.121, *p* = 0.432; all patients: r = −0.055, *p* = 0.613).

**Figure 2:**
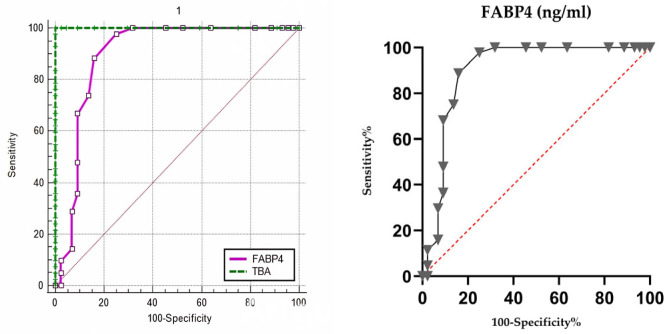
Receiver operating characteristic (ROC) curve for FABP4 distinguishing ICP from healthy pregnancies and comparison of FABP4 and total bile acid. AUC = 0.899 (95% CI: 0.816–0.953, *p* < 0.001), with an optimal cut-off of 3.0 ng/mL providing 90% sensitivity and 84% specificity for ICP diagnosis. The red dashed line indicates the line of no discrimination (AUC = 0.5). Triangle symbols represent different cutoff values corresponding to sensitivity–specificity pairs on the ROC curves.

Receiver operating characteristic (ROC) analysis showed that maternal serum FABP4 had excellent diagnostic accuracy in distinguishing ICP from healthy pregnancies, with an area under the curve (AUC) of 0.899 (95% CI: 0.816–0.953, *p* < 0.001). A cut-off value of >3.0 ng/mL yielded a sensitivity of 90% and a specificity of 84% for ICP diagnosis (Figure 2). Detailed diagnostic and predictive performance metrics for various FABP4 thresholds are provided in Supplementary Table S1.

**Figure 3:**
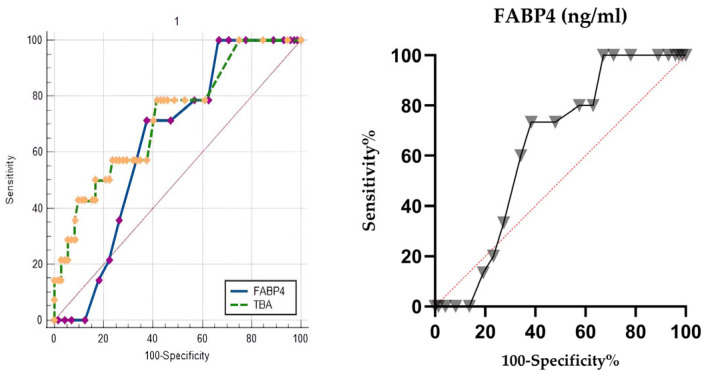
Receiver operating characteristic (ROC) curve for FABP4 predicting composite neonatal outcomes in the total cohort (ICP group and Control group) and comparison of FABP4 and total bile acid. FABP4: AUC = 0.634 (95% CI: 0.525–0.734, *p* = 0.032). A cut-off value of 3.2 ng/mL yielded 73% sensitivity and 62% specificity. No statistically significant difference was found between TBA and FABP4 for CNO prediction in the ROC comparison analysis (*p* = 0.189). The red dashed line indicates the line of no discrimination (AUC = 0.5). Rhombus and triangle symbols represent different cutoff values corresponding to sensitivity–specificity pairs on the ROC curves.

**Figure 4:**
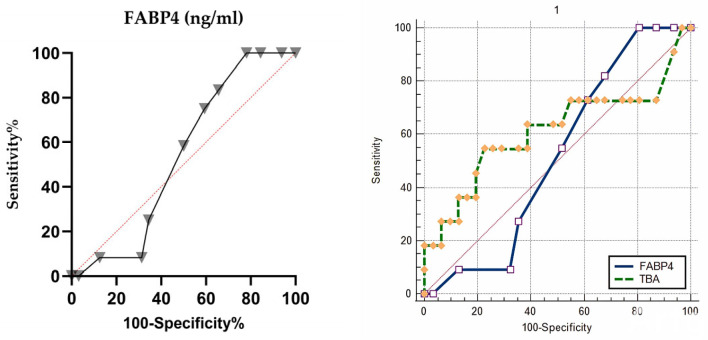
Receiver operating characteristic (ROC) curve for maternal serum FABP4 predicting composite neonatal outcomes in the ICP subgroup and comparison of FABP4 and total bile acid. The area under the curve (AUC) was 0.535 (95% CI: 0.379–0.687, *p* = 0.685), indicating limited predictive performance for adverse neonatal outcomes within the ICP cohort. No statistically significant difference was found between TBA and FABP4 for CNO prediction in the ROC comparison analysis (*p* = 0.513). The red dashed line indicates the line of no discrimination (AUC = 0.5). Rhombus, square, and triangle symbols represent different cutoff values corresponding to sensitivity–specificity pairs on the ROC curves.

In predicting composite neonatal outcomes in the entire cohort, FABP4 showed moderate discriminatory power with an AUC of 0.634 (95% CI: 0.525–0.734, *p* = 0.032). The optimal cut-off value (>3.2 ng/mL) yielded a sensitivity of 73% and a specificity of 62% (Figure 3). However, when the analysis was limited to the ICP subgroup, FABP4 did not reliably predict negative neonatal outcomes (AUC = 0.535, 95% CI: 0.379–0.687, *p* = 0.685) (Figure 4).

## 4. Discussion

In this prospective study, we found significantly elevated maternal serum FABP4 levels in pregnancies complicated by ICP compared with healthy controls, with excellent diagnostic accuracy (AUC = 0.899). These findings build on previous data about the metabolic and inflammatory aspects of ICP and suggest that FABP4, a multifunctional adipokine involved in lipid transport and immune regulation, may serve as a clinically useful biomarker reflecting the metabolic dysregulation characteristic of cholestatic pregnancies.

FABP4, primarily secreted by adipocytes and macrophages, regulates lipid homeostasis, insulin sensitivity, and inflammatory cascades [14]. During normal pregnancy, its circulating concentrations rise moderately, paralleling metabolic adaptations; however, excessive elevations—as observed in gestational diabetes mellitus and preeclampsia—reflect maladaptive lipid metabolism and endothelial dysfunction [14]. Our data indicate that ICP may share similar pathogenic mechanisms, supporting the view that it represents not only a hepatobiliary disorder but also a systemic metabolic perturbation. In addition, our results show that FABP4 levels have diagnostic value for ICP even without an increase in ESAIX levels, which indicate endothelial damage. It is understood that in the early stages of ICP, before endothelial damage occurs due to inflammatory processes caused by lipid metabolism disorders, FABP4 blood levels increase significantly.

Recent multi-omics research highlights the multifactorial pathophysiology of ICP. Proteomic and metabolomic profiling have identified disruptions in lipid oxidation, peroxisomal β-oxidation, and PPAR signaling [15,16,17]. Zeng et al. demonstrated upregulation of APOA2 and PPARα/RXRα activation in ICP placentas, linking lipid transport and nuclear receptor regulation to disease pathogenesis [15]. Similarly, Dong et al. reported altered levels of ACOX1 and L-palmitoylcarnitine, both involved in fatty acid oxidation [16]. Our observation of elevated FABP4—a lipid chaperone regulating PPAR activity—fits coherently within this molecular network, suggesting a common axis of dysregulated lipid signaling, oxidative stress, and inflammatory activation.

Additionally, recent transcriptomic and small RNA studies highlight the role of non-coding RNAs in metabolic regulation. Yang et al. demonstrated that dysregulated tRNA-derived fragments impair fatty acid degradation in ICP, further emphasizing the central role of lipid metabolism in disease etiology [18]. Taken together, FABP4 elevation may serve as both a marker and a mediator within the broader lipid–inflammation–bile acid axis.

The traditional view of ICP focuses on bile acid accumulation and hormonal modulation. However, bile acids are potent metabolic regulators that act through FXR and TGR5 receptors. Tang et al. recently showed that gut microbiota—particularly Bacteroides fragilis—can induce cholestasis by inhibiting FXR and altering bile acid metabolism [19]. This gut–liver–metabolic crosstalk supports our hypothesis that elevated FABP4 reflects systemic lipid and inflammatory imbalance rather than isolated hepatocellular injury. Additionally, experimental hypercholanemia in mice suppresses pregnancy-associated adipose expansion and stimulates pro-inflammatory responses [20], suggesting that bile acid overload could secondarily increase FABP4 release from adipose or trophoblastic tissues. Consistent with our findings, Ozkan et al. recently reported that systemic inflammatory indices such as the systemic immune-inflammation index (SII) and neutrophil-to-lymphocyte ratio (NLR) were significantly elevated in women with ICP, indicating an underlying low-grade inflammatory state [21]. These data further support the concept that metabolic and inflammatory dysregulation coexist in ICP, reinforcing FABP4’s potential role at the intersection of lipid metabolism and immune activation.

Adipokines, including leptin, apelin, and adiponectin, have also been implicated in ICP, showing altered serum profiles correlated with disease severity [22]. These molecules share overlapping pathways with FABP4 in regulating energy metabolism and vascular tone. The convergence of elevated FABP4 and dysregulated adipokines supports the concept of ICP as a metabo-inflammatory disorder characterized by cross-talk among hepatic, adipose, and placental tissues.

FABP4’s excellent diagnostic accuracy (AUC = 0.899) compares favorably with other emerging biomarkers such as glycocholic acid and L-palmitoylcarnitine identified through metabolomics (AUC = 0.89–0.99) [16], and urine metabolites like 3-hydroxypropionic acid (AUC = 0.92) [23]. Unlike bile acid testing, FABP4 measurement via ELISA is rapid and widely available, offering potential for early detection, particularly in atypical or subclinical cases. However, its prognostic capacity for neonatal outcomes was limited, likely reflecting the multifactorial determinants of fetal compromise—including bile acid-induced arrhythmogenicity, placental hypoxia, and vascular dysfunction—rather than maternal metabolic stress alone [24].

While our study did not include a detailed investigation of the mechanism of action of FABP4, it highlighted the potential influence of FABP4 on the development of ICP, considering both maternal and neonatal outcomes due to the complex nature of ICP pathophysiology. However, we concluded that FABP4 does not play an effective role in evaluating neonatal outcomes. When all patients were evaluated, the correlation between FABP4 and the timing of delivery was not an indicator of neonatal well-being or favorable perinatal outcomes, but rather an indirect representation of FABP4’s diagnostic value. In patients diagnosed with ICP, delivery occurred earlier than in the control group, and FABP4 levels were higher. The absence of a correlation between FABP4 and the timing of delivery in the ICP group, but the negative correlation observed when the control group was included, indicates that FABP4 is not only a diagnostic marker for ICP but also a molecule that may influence clinicians’ decisions regarding neonatal outcomes.

The combined evaluation of FABP4 and TBA measurements may be a valuable tool for diagnosing ICP and achieving optimal neonatal outcomes. This approach could influence delivery timing and follow-up protocols. In cohorts with longitudinal series of FABP4 and TBA measurements, adequate sample sizes, and diverse centers, it is possible to develop diagnostic models that improve neonatal outcomes. Since prognosis cannot be determined by decreased TBA levels after ursodeoxycholic acid treatment for ICP, and follow-up protocols and delivery timing are currently based on the TBA level at diagnosis, combining TBA and FABP4 may provide clinical benefits.

Our study’s strengths include its prospective design, standardized sampling, and well-matched controls. Limitations include the relatively small, single-center cohort, cross-sectional measurement of FABP4, and absence of mechanistic assays. Further studies should validate FABP4 dynamics across gestation and integrate multi-omics data—proteomic, metabolomic, and microbiome signatures—to establish composite predictive models. Conducting these studies at multiple centers with larger sample sizes will increase their reliability and address the shortcomings of our study in this regard. As studies suggest that the diagnostic value of various diseases in pregnancy [25] can be improved by using combined markers, combining FABP4 with omics-based biomarkers (such as APOA2, ACOX1, or microbial-derived bile acid profiles) may increase diagnostic sensitivity and provide a better understanding of lipid-bile acid homeostasis.

## 5. Conclusions

Overall, our findings identify FABP4 as a novel biomarker with high diagnostic but limited prognostic value in ICP. In neonatal outcome assessment, the nature of ICP inherently introduces confounding factors because of differences in delivery times between groups. However, even when evaluating only patients diagnosed with ICP, FABP4 does not have prognostic predictive value for neonatal outcomes. Integrating FABP4 into the emerging framework of metabolic–inflammatory pathogenesis—supported by recent proteomic, transcriptomic, and microbiome evidence—suggests that ICP reflects systemic dysregulation of lipid and bile acid metabolism. Future translational efforts should focus on elucidating FABP4-mediated pathways and exploring its interactions with PPAR, FXR, and inflammatory signaling to improve early detection and therapeutic targeting of ICP.

## Figures and Tables

**Table 1:** Demographic characteristics and clinical features of the groups.

	Control Group	ICP Group	*p*-Value
Maternal age (year) (mean ± SD)	27.5 (25.0–32.8)	27.0 (25.0–30.8)	0.469
Gestational weight gain (kg) (median, Q_1_–Q_3_)	11 (9–12)	10 (8–12)	0.243
BMI at sampling (kg/m^2^) (mean ± SD)	26.8 ± 3.40	27.9 ± 3.54	0.142
Gravidy, median (Q_1_–Q_3_)	2 (1–3)	2 (1–2)	0.055
Parity, median (Q_1_–Q_3_)	1 (0–2)	0 (1–0)	0.031
Miscarriage, median (Q_1_–Q_3_)	0 (0–0)	0 (0–0)	0.868
Gestational age at the day of sampling (week)	35 (31–37)	35 (31–36)	0.924
Systolic blood pressure (mmHg) median (Q_1_–Q_3_)	110 (100–110)	130 (120–130)	<0.001
Diastolic blood pressure (mmHg) median (Q_1_–Q_3_)	70 (60–74)	70 (69–80)	0.008

Data are presented as mean ± SD or median (Q_1_–Q_3_), as appropriate. Differences between groups were assessed using independent-samples *t*-test or Mann–Whitney U test for continuous variables and chi-square or Fisher’s exact test for categorical variables. ICP: intrahepatic cholestasis of pregnancy; BMI: Body mass index.

**Table 2:** Comparison of hematological and biochemical parameters between women with ICP and healthy controls.

	Control Group	ICP Group	*p*-Value
WBC (10^3^/μL)	9.15 ± 2.002	10.05 ± 2.460	0.064
Hemoglobin (g/dL)	11.6 ± 1.323	11.8 ± 1.318	0.573
Hematocrit (%)	36.4 (33.6–38.8)	36.5 (33.6–38.7)	0.950
Platelets (10^3^/μL)	243 ± 70.2	249 ± 60.8	0.668
Neutrophils (×10^3^/µL)	6.67 (5.32–8.08)	7.42 (5.86–8.51)	0.109
Lymphocytes (×10^3^/µL)	1.72 (1.54–2.01)	1.77 (1.35–2.30)	0.723
ALT (U/L)	11 (8–15)	74 (43–214)	<0.001
AST (U/L)	15 (13–19)	59 (32–112)	<0.001
Creatinine (mg/dL)	0.50 (0.46–0.56)	0.47 (0.42–0.50)	0.056
BUN (mg/dL)	14 (11–18)	14 (7–18)	0.363
ESAIX	0.35 (0.25–0.45)	0.35 (0.26–0.55)	0.376
Fasting glucose (mg/dL)	81 (72–92)	87 (72–99)	0.214
TBA (μmol/L)	4 (2–5)	28 (20–45)	<0.001
aPTT (s)	27.0 (25.0–28.4)	26.0 (23.5–29.2)	0.329
PT (s)	7.80 (7.61–8.20)	8.04 (7.13–10.50)	0.122
INR	0.89 (0.87–0.92)	0.92 (0.90–1.01)	0.007
Fibrinogen (mg/dL)	453 (418–500)	571 (507–630)	<0.001
Systolic blood pressure (mmHg)	110 (100–110)	130 (120–130)	<0.001
Diastolic blood pressure (mmHg)	70 (60–74)	70 (69–80)	0.008
FABP4 (ng/mL)	2.40 (2.00–2.95)	3.60 (3.25–4.20)	<0.001
*<34 weeks (n = 18)*	2.60 (2.35–3.10)	3.60 (3.15–4.20)	0.001
*34–37 weeks (n = 19)*	2.06 ± 0.786	3.80 ± 0.447	<0.001
*≥37 weeks (n = 7)*	2.57 ± 0.725	3.94 ± 0.629	0.003

Data are expressed as mean ± SD or median (Q_1_–Q_3_). ICP: intrahepatic cholestasis of pregnancy; WBC: White Blood Cell; BUN: Blood Urea Nitrogen; ESAIX, Endothelial Activation and Stress Index; ALT: alanine aminotransferase; AST: aspartate aminotransferase; TBA: total bile acid; aPTT: activated partial thromboplastin time; PT: prothrombin time; INR: international normalized ratio; FABP4: fatty acid-binding protein 4. Statistical significance set at *p* < 0.05.

**Table 3:** Perinatal outcomes of pregnancies with ICP compared to controls.

	Control Group	ICP Group	*p*-Value
Gestational week at the day of delivery	39 (38–39)	37 (36–37)	<0.001
Birth weight (g); median (Q_1_–Q_3_)	3100 (2940–3260)	2775 (2540–3160)	0.002
APGAR Score 1st minute, median (Q_1_–Q_3_)	9 (9–9)	9 (8–9)	0.036
APGAR Score 5th minute, median (Q_1_–Q_3_)	10 (10–10)	10 (9–10)	0.072
Type of delivery, *n* (%)			
*Cesarean section*	21 (47.7)	22 (50.0)	N.S.
*Vaginal delivery*	23 (52.3)	22 (50.0)	
NICU admission, *n* (%)	2 (4.5)	7 (15.9)	0.157
Poor CNO, *n* (%)	3 (6.8)	12 (27.3)	0.023

Data are shown as median (Q_1_–Q_3_) or *n* (%). ICP: intrahepatic cholestasis of pregnancy; NICU: Neonatal Intensive Care Unit; CNO: composite neonatal outcome, defined as the presence of at least one of the following—preterm birth < 34 weeks, low birth weight (<2500 g), 5 min APGAR score < 7, or neonatal intensive care unit (NICU) admission. N.S.—Not Significant.

**Table 4:** Correlations between maternal serum FABP4 levels and maternal–perinatal parameters.

	All Patients		Only ICP Group	
	*r*	*p*	*r*	*p*
Maternal age	−0.021	0.848 *	0.055	0.722 **
BMI	−0.004	0.969 **	0.046	0.768 **
Gestational weight gain	−0.144	0.183 *	−0.177	0.448 *
Parity	−0.203	0.058 *	−0.143	0.355 *
Gestational age at delivery	−0.430	<0.001 *	−0.109	0.483 *
Gestational age at blood sample taken	−0.055	0.613	0.121	0.432
Birth weight	−0.125	0.246 *	0.085	0.585 **
APGAR score at 1st minute	−0.144	0.179 *	−0.060	0.698 *
APGAR score at 5th minute	−0.125	0.247 *	−0.105	0.497 *
TBA	0.594	<0.001 **	−0.041	0.798 **

*r*: correlation coefficient; *p*: significance value. Spearman’s correlation used for nonparametric data (*), Pearson’s for parametric data (**). ICP: intrahepatic cholestasis of pregnancy; BMI: Body mass index; TBA: total bile acid.

## Data Availability

The data presented in this study are available on request from the corresponding author due to data protection.

## Supplementary Materials

The following supporting information can be downloaded at: https://www.mdpi.com/article/10.3390/diagnostics16040525/s1. Table S1: Receiver operating characteristic (ROC) analysis showing optimal FABP4 cut-off values for the diagnosis of ICP and prediction of poor composite neonatal outcomes.

## Abbreviations

The abbreviations used throughout the manuscript are listed below:

FABP4	Fatty acid-binding protein 4
ICP	Intrahepatic cholestasis of pregnancy
ELISA	Enzyme-linked immunosorbent assay
ROC	Receiver operating characteristic
AUC	Area under the curve
CI	Confidence interval
CNO	Composite neonatal outcome
GDM	Gestational diabetes mellitus
PPARs	Peroxisome proliferator-activated receptors
CV	Coefficients of variation
ESAIX	Endothelial Activation and Stress Index
AST	Aspartate aminotransferase
ALT	Alanine aminotransferase
TBA	Total bile acid
BUN	Blood urea nitrogen
WBC	White Blood Cell
aPTT	Activated partial thromboplastin time
PT	Prothrombin time
INR	International normalized ratio
BMI	Body mass index
