# Supplementary material for: Serum Fatty Acid-Binding Protein 4: A Potential Diagnostic Marker Linking Lipid Metabolism and Inflammation in Intrahepatic Cholestasis of Pregnancy
Source: Diagnostics (Basel). 2026 Feb 10;16(4):525. doi: 10.3390/diagnostics16040525 (PMC12939078; doi:10.3390/diagnostics16040525)
Supplement: Supplementary file 1 [file diagnostics-16-00525-s001.zip › diagnostics-4037448-supplementary.pdf]

Supplementary Table S1. Receiver operating characteristic (ROC) analysis showing optimal FABP4 cut-off values for the diagnosis of ICP and prediction of poor composite neonatal outcomes.

	AUC	CI (95%)	*p*	cut-off	Sensitivity (%)	Specificity (%)	+LR	-LR
**ICP diagnosis**	0.899	0.816-0.953	**<0.001**	>2.8	98	75	3.91	0.03
				>3.0	90	84	5.57	0.14
				>3.4	68	90	7.50	0.35
**CNO**	0.634	0.525-0.734	**0.032**	>3.2	73	62	1.91	0.43
				>2.6	90	37	1.27	0.54
(All patients)				>4.2	-	90	-	1.16
**CNO**	0.535	0.379-0.687	0.685	≤3.2	8	69	0.27	1.33
				≤4.0	90	34	1.27	0.48
(ICP subgroup only)				≤3.0	8	90	0.67	1.05

AUC: area under the curve; CI: confidence interval; +LR: positive likelihood ratio; −LR: negative likelihood ratio; CNO: composite neonatal outcome; ICP: intrahepatic cholestasis of pregnancy. Statistically significant p-values are shown in bold.
